# Building Endogenous Capacity for the Management of Neglected Tropical Diseases in Africa: The Pioneering Role of ICIPE

**DOI:** 10.1371/journal.pntd.0002687

**Published:** 2014-05-15

**Authors:** Daniel K. Masiga, Lilian Igweta, Rajinder Saini, James P. Ochieng'-Odero, Christian Borgemeister

**Affiliations:** 1 International Centre of Insect Physiology and Ecology, ICIPE, Nairobi, Kenya; 2 Consortium for National Health Research (CNHR), Nairobi, Kenya; Yale School of Public Health, United States of America

## Introduction

### Institutionalizing research and training for the management of neglected tropical diseases (NTDs) through establishment of a world-class research centre

In a recent article titled “Neglected No Longer: New Progress on NTDs,” Richard Hartfield argues that NTDs are now mainstream because they are on the agenda of three key policy forums―the African Union (AU), the World Health Assembly (WHA), and the Organization of American States (OAS) (http://www.impatientoptimists.org/Posts/2013/08/Making-Progress-on-NTDs). While this is good news, these pathogens still retard the development and health of rural communities, especially in Africa. Several NTDs are caused by pathogens transmitted by arthropod vectors, principally mosquitoes, tsetse flies, and ticks. New initiatives bring welcome attention and resources, which may finally lead to the control of diseases like sleeping sickness that were reported to have been controlled during decades of intensive control [1]. Yet to get here, a point at which talk of elimination and eradication of some NTDs is possible, has taken years of building African capacity, with considerable support from donors, institutions, and individuals. This historical review highlights the contribution of the International Centre for Insect Physiology and Ecology (ICIPE) to knowledge creation and the development of tools and approaches for control of arthropod-transmitted NTDs. We discuss the establishment and growth of the Centre and highlight the vision and research leadership of its founding director, the late Professor Thomas Risley Odhiambo (fondly referred to as TRO), and his successors in establishing this world-class research institution.

### Thomas Risley Odhiambo: A philosophy and lifelong commitment to a science-led vision for development

The genesis of ICIPE can be traced to TRO's seminal paper published in the November 1967 issue of *Science*, describing how science could spur development in East Africa [2]. He attributed the predicament of science in the region to poor administration, inadequately trained human resources, and the absence of a science policy related to national or regional development, among other reasons. This thinking motivated mobilization of national and international support that led to the creation of ICIPE. At the forefront was Carl Djerassi who, based on his outstanding work in Mexico on natural products, had made a compelling case for the establishment of “centres of excellence in developing countries to develop local scientific capabilities”[3].

ICIPE was formally established in Nairobi in 1970 as an international institution of advanced research with the goal of ensuring food security and better health for humankind and their livestock while protecting the environment. Initial research work was organized under four sections: (1) Insect ecology and genetics, (2) insect sensory physiology and behaviour (establishing the first electrophysiology laboratory in Africa), (3) insect hormones and chemistry, and (4) biophysics of insects. Five insect species were initially selected because of their critical importance to Africa, three of them—tsetse, ticks, and mosquito vectors of the Yellow Fever Virus—vectors of NTDs. Research capacity was initially developed and strengthened under the leadership of visiting directors of research while mentoring African researchers (e.g., the staff of national research organizations, university staff, and PhD and MSc scholars).

**Figure 1:**
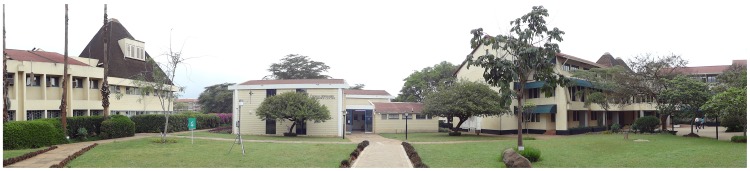
A panoramic view of ICIPE's Duduville campus, the Centre's headquarters, in Nairobi, showing (from left to right): the R&D building, the Thomas Odhiambo conference centre in the middle, and the administration building to the right.

TRO's ability to raise funds, negotiate, and organize was further demonstrated by his influence in the formation of the African Academy of Sciences (AAS) in 1985, for which he also served as President from 1986 to 1999, and by helping to set up the African Foundation for Research and Development (AFRAND) (http://www.uia.be/s/or/en/1100049901). He received several awards in recognition of his scientific and other achievements, including the African Prize for Leadership for the Sustainable End of Hunger (1987), jointly with the then president of Senegal, Abdou Diouf, from US President Ronald Reagan [4]. His negotiating skills brought the then president of Kenya, Daniel arap Moi, to ICIPE's Nairobi headquarters (Figure 1) and the campus at Mbita Point in western Kenya (now named the Thomas Odhiambo Campus [TOC]) during the official inauguration events in 1986. It is on such foundations that the African Union has in recent years determinedly promoted science and technology as drivers of innovation and development (http://hrst.au.int/en/).

### Hans Rudolf Herren: A new research paradigm in support of human and animal health

TRO was succeeded as director-general (DG) in 1994 by Swiss scientist Dr. Hans Rudolf Herren, who led the Centre until 2005. Hans came from the background of having led a campaign to control the cassava mealy bug (*Phenacoccus manihoti*) using its exotic natural enemy *Epidinocarsis lopezi*, for which he received several awards, including the 1995 World Food Prize [5]. He reorganized the research activities at ICIPE into the 4-H paradigm (Human, Animal, Plant, and Environmental Health). As a result, research in diseases such as trypanosomiasis and leishmaniasis was undertaken mainly in Animal and Human Health divisions.

### Developing “smart” tools to manage vectors of NTDs

**Figure 2:**
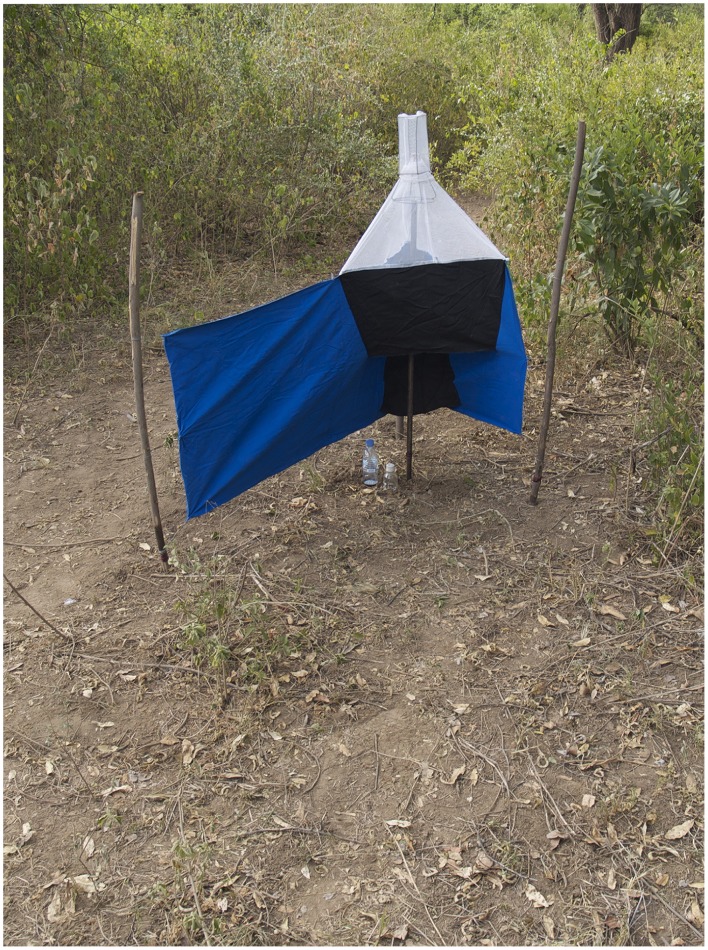
A Ngu trap (named after Nguruman, where it was developed) developed for savannah species of tsetse flies. The baited trap is based on both visual (shape and colour) and olfactory cues (e.g., using cow urine and acetone).

**Figure 3:**
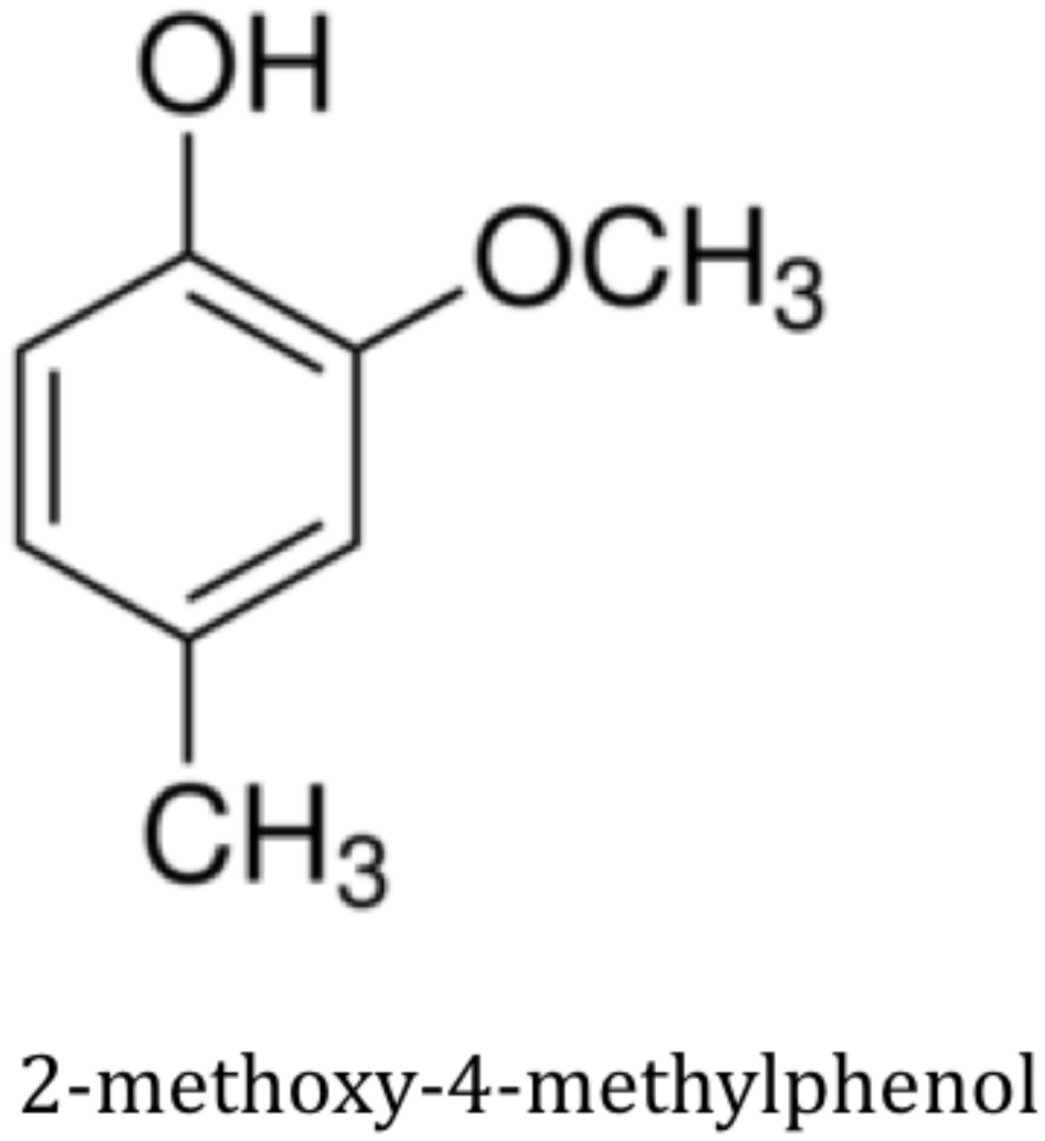
The structure of 2-methoxy-4-methylphenol, a compound based on a naturally occurring repellent from the waterbuck (*K. defassa*).

**Figure 4:**
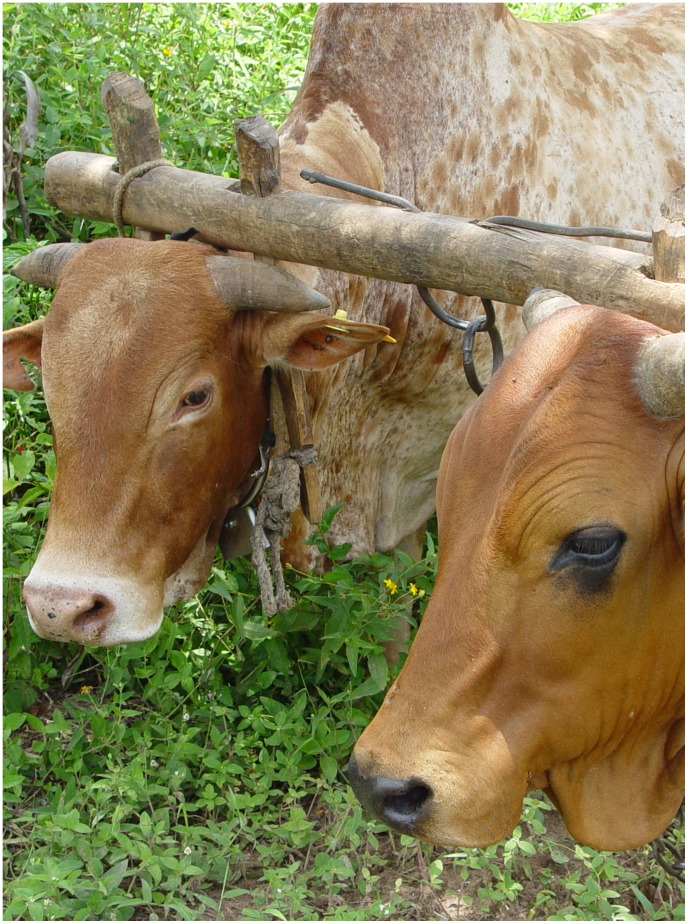
Bulls plough a field at the Kenya Coast. The collars around their necks contain a device that slowly releases a blend of tsetse fly repellents to deter tsetse flies from feeding. This provides the animals with a significant level of protection against infection with trypanosomes.

The discovery of components of the chemical language that enable communication among insects and their environment is a well-travelled path at ICIPE. Right from ICIPE's establishment, the basic science of how insects interact within their environment was prioritized. Among the eminent scientists who left their lasting footprint on the scientific legacy of the Centre in insect communication was the first visiting research director, Martin Lüscher, who is also well-known for having coined the word “pheromone” [6]. Vector ecology became a prime driver of research and innovation for disease management as scientists sought to identify visual and olfactory cues and how these could be used to exploit insect behaviour for developing simple technologies for control. Initial outputs of this investment included the Ngu trap (named after ICIPE's field station of Nguruman, where it was developed) for the savannah species of tsetse (Figure 2) [7], which was based on bioassays that defined such attributes as trap colour, shape, and size. The efficiency of these traps (“artificial cows”) was enhanced several fold if the chemicals that attract tsetse to their hosts were placed next to them. Such chemical cues were identified from skin secretions and components of the urine of animal blood meal sources, such as buffalo and oxen [6]–[13]. Odour-baited traps and screens can be used to suppress fly populations by 99% [14], and in their simplicity these tools lend themselves to use by local communities. Experiments conducted at our Mbita Point field site and with collaborators in West Africa have shown that small targets or screens offer a cost-effective and efficient tool for control of the riverine species *Glossina fuscipes fuscipes* in East Africa [15] and other “Palpalis group” flies in West Africa [16]. However, these devices are inappropriate for nomadic herders, who are frequently on the move in search of new grazing fields. A mobile tsetse control technology is more suitable, a realization that led ICIPE scientists to consider repellent technology that would move together with cattle herds, and they started looking at both synthetic and animal-derived chemicals. It was shown that although present in tsetse fly habitats, waterbuck (*Kobus defassa*) were rarely fed on by tsetse. These studies led to the identification of a synthetic tsetse repellent 2-methoxy-4-methylphenol (Figure 3) (ICIPE patent #KE/P/10/001179) [17] and a 5-constituent waterbuck repellent blend (ICIPE patent pending). Deployment as a slowly released formulation in a collar worn around individual cattle's necks is under field evaluation (Figure 4). While improvements in its efficiency are likely, this technology provides substantial protection to cattle either in “push” mode or when used in conjunction with baited traps in a “push-pull” strategy [18]. The odour-baited traps and targets and repellent technology are good examples of how basic research can lead to the development of simple technologies with substantial benefits for the food security and livelihoods of smallholder communities.

Research in the field of tsetse flies and trypanosomiasis has been boosted by the publication of the genome of *G. morsitans morsitans* by the International Glossina Genome Initiative (IGGI) [19], [20] with the participation of ICIPE scientists. We can now explain the genetic basis of some ecological observations, such as attraction to hosts for feeding, for example by the observation of greater investment in receptors for CO_2_ by tsetse flies [21], and how the flies meet their needs for homeostasis for many physiological functions, using aquaporins to move fluids across membranes [22]. Further investment in bioinformatics and functional genomics will be required to optimally use the publicly available genomic resources.

### Targeting the sandfly vector of leishmaniasis

The Biosystematics Support Unit at ICIPE hosts many slide mounts of sandflies (phlebotomine vectors of leishmaniasis), which shows the amount of work that went into surveys to describe sandfly biodiversity and disease epidemiology [23]–[32]. This and more recent work have contributed considerably to understanding the ecology and vector status of sandflies in East Africa [32]–[38] and the efficacy of biological agents such as fungal pathogens as part of an integrated control package [33]. It is as a result of discoveries such as this that renewed interest in sandfly research has been generated after years of decline.

### Christian Borgemeister: Expanding ICIPE's research portfolio of NTDs

ICIPE's third DG, Professor Christian Borgemeister (2005–2013), has extended the benefits of the 4-H paradigm by consolidating thematic teams, with the Integrated Vector and Disease Management (IVDM) cluster incorporating Animal and Human Health functions, and by bringing in additional capacities in geospatial sciences. While expanding many research streams, Borgemeister has spearheaded efforts to develop the necessary infrastructure to adequately address arthropod-transmitted viral (arboviral) and bacterial pathogens, including Rift Valley fever (RVF). The newly established Martin Lüscher Emerging Infectious Diseases Laboratory (MLEID), which has an insectary and a Biosafety Level 3 (BSL3) facility that offer appropriate containment, is the centrepiece for these activities, important for studies of arboviruses vectored by mosquitoes and ticks. The MLEID has also enhanced capacity building through postgraduate-level training and short-term training for students from several countries.

Growing ICIPE's research portfolio in this way has allowed the institute to advance knowledge in vector ecology along the path toward developing vector control strategies for reducing the threat and impact of emerging infectious diseases (EIDs). These studies include disease epidemiology [39]–[41], the development of new attractants and improvements in existing tools [42], [43] for control of mosquito vectors. We are undertaking this research and development (R&D) in multi-institutional collaborations to more efficiently utilize complementary expertise and resources (http://www.icipe.org/avid/; http://www.icipe.org/cernvec). In 2012, ICIPE was designated as a Food and Agriculture Organization of the United Nations (FAO) Reference Centre for vectors and vector-borne animal diseases (tsetse flies and animal trypanosomiasis and arthropod-transmitted viral human pathogens) in recognition of ICIPE's “attainment of scientific, technical, and policy standing and its commitment to strengthen capacity development in those areas relevant to FAO's mandate.”

### Building capacity for research and management of NTDs: The African Regional Postgraduate Programme in Insect Science (ARPPIS) and the Dissertation Research Internship Programme (DRIP)

ARPPIS (www.icipe.org/arppis) is a collaborative programme between ICIPE and African universities, which aims to (1) build capacity on the continent and (2) generate knowledge in innovative insect science, thus contributing to the reduction of poverty and food insecurity. Since its launch in 1983, ARPPIS has trained more than 250 PhD-level scientists and in excess of 300 at the level of MSc by offering fully sponsored fellowships, mostly through the three regional centres: the University of Ghana at Legon, Addis Ababa University, and the University of Zimbabwe in Harare. DRIP complements ARPPIS by enabling students not eligible for ARPPIS fellowships to benefit from ICIPE's research mentorship. This has particularly benefited the researchers from government institutions (such as national agricultural and health research centres from various countries), who often cannot enrol in full-time study. These training programmes have led to considerable output in terms of theses, publications, and new knowledge and tools. More than 30 doctoral theses on NTDs and more than double this number at the MSc level have been published. It is notable that some of the scholars have taken key leadership positions in their countries (e.g., Professor J. H. Pen-Mogi Nyeko as vice chancellor of Gulu University in Uganda and Professor M. Imbuga as vice chancellor of Jomo Kenyatta University of Agriculture and Technology in Kenya). During an interview carried out for an ARPPIS alumni tracer study in 2012, Professor Nyeko observed that the ARPPIS program is particularly useful in building teaching capacity at universities as it “trains PhD candidates who go back to teach others, thereby creating a cycle of knowledge recreation.” He also observed that this is but a small step in alleviating a great sea of need in Africa.

From knowledge to processes and tools, ICIPE continues to leave a footprint on reducing the effect of NTDs in Africa and beyond. However, there have been challenges, such as funding fluctuations and limited endogenous in-country support, which have not facilitated continuous scientific enquiry. The ebbs and flows of leishmaniasis research is a case in point. While it is difficult to quantify the contribution ICIPE has made to R&D associated with NTDs, the Centre's footprint on the African continent is large. We celebrate this and believe the foundations for growth and impact have been laid.

A list of publications from ICIPE scientists and students on NTDs, categorized according to disease, is provided as supplementary material.

## Supporting Information

Supporting Information S1 ICIPE's publications on NTDs. Peer-reviewed publications by ICIPE scientists and collaborators on neglected tropical diseases. Since ICIPE's inception in 1970, more than 200 peer-reviewed publications have been published, an output that exceeds 5 per year. As a reflection of earlier years, more was done on African trypanosomiasis than other NTDs.
(DOC)
