# Supplementary material for: Building Endogenous Capacity for the Management of Neglected Tropical Diseases in Africa: The Pioneering Role of ICIPE
Source: PLoS Negl Trop Dis. 2014 May 15;8(5):e2687. doi: 10.1371/journal.pntd.0002687 (PMC4022455; doi:10.1371/journal.pntd.0002687)
Supplement: Supporting Information S1 — ICIPE's publications on NTDs. Peer-reviewed publications by ICIPE scientists and collaborators on neglected tropical diseases. Since ICIPE's inception in 1970, more than 200 peer-reviewed publications have been published, an output that exceeds 5 per year. As a reflection of earlier years, more was done on African trypanosomiasis than other NTDs. (DOC) [file pntd.0002687.s001.doc]

# Publications on Neglected Tropical Diseases (NTDs) by *icipe* scientists and collaborators. These are presented in three categories: African trypanosomiasis; Leishmaniasis, Arboviruses and Ticks (because ticks vector many pathogens, some not NTDs).

## African trypanosomiasis

1.	Abubakah L.; Osir, E.O.; Imbuga, M.O., 1995. Properties of a blood-meal-induced midgut lectin from the tsetse fly *Glossina morsitans*. *Parasitol Res*, 81: 271–275.

2.	Abubakah, L.U.; Zimba, G.; Wells, C.; Mulaa, F.; Osir, E.O., 2003. Evidence for the involvement of a tsetse midgut lectin-trypsin complex in differentiation of bloodstream-form trypanosomes. *Insect Sci Applic* 23: 197–205.

3.	Abubakar LU, Bulimo WD, Mulaa FJ, Osir EO. Molecular characterization of a tsetse fly midgut proteolytic lectin that mediates differentiation of African trypanosomes. *Insect Biochem Mol Biol*. 2006 Apr;36(4):344-52. Epub 2006 Jan 19. PubMed PMID: 16551548.

4.	Amin, D.N.; Kamita, S.G.; Muluvi, G.M.; Machuka, J.; Hammock, B.D.; Osir, E.O., 2006. Glossina proteolytic lectin does not require a carbohydrate moiety for enzymatic or trypanosome-transforming activities. *J. Med Entomol.* 43: 301–308.

5.	Burugu, M.W.; Mbatia, B.N.; Osir, E.O.; Kenya, E.U.; Abubakar, L.U., 2008. The| Glossina proteolytic lectin (Gpl) gene is expressed only in members of Glossina species. *Int J Trop Insect Sci*. 27: 145–151.

6.	Brightwell R, Dransfield RD, Kyorku C., 1991. Development of a low-cost tsetse trap and odour baits for *Glossina pallidipes* and *G. longipennis* in Kenya. *Med Vet Entomol*. 5): 153-64. PubMed PMID: 1768909.

7.	Brightwell R, Dransfield R., 1997. Odour attractants for tsetse: Glossina austeni, *G.brevipalpis* and *G.swynnertoni*. *Med Vet Entomol*. 11: 297-9. PubMed PMID: 9330263.

8.	Challier, A. and Gouteux, J.P., 1980. Ecology and Epidemilogical importance of Glossina Palpalis in the Ivory Coast forest zone. *Insect Sci Applic* 1: 77-83.

9.	Denlinger, D. L., Saini, R. K. and Chaudhury, M. F. B., 1983. Parturition in the tsetse fly *Glossina morsitans morsitans*: Pattern of activity, sound production and evidence for control by the mother’s brain. *J. Insect Physiol*. 29: 715-721.

10.	Den Otter, C. J. and Saini, R. K., 1985. Pheromone perception in the tsetse fly *Glossina morsitans morsitans*. *Entomol. Exp. Appl*. 39, 155 - 161.

11.	Dransfield, R.D., Brightwell, R., Kiilu, J., Chaudhury, M.F., Adabie, D.A., 1989. Size and mortality rates of *Glossina pallidipes* in the semi-arid zone of southwestern Kenya. *Med Vet Entomol*. 3: 83-95. PubMed PMID: 2519651.

12.	Dransfield RD, Brightwell R, 1989. Problems of field testing theoretical models: a case study. *Ann Soc Belg Med Trop*. 69 Suppl 1:147-54; discussion 212-4. PubMed PMID: 2802817.

13.	Dransfield RD, Williams BG, Brightwell R., 1991. Control of tsetse flies and trypanosomiasis: Myth or reality? *Parasitol Today*. 7: 287-91. PubMed PMID: 15463392.

14.	Dransfield RD, Brightwell R, 2001. Trap efficiency for *Glossina pallidipes* (Diptera: Glossinidae) at Nguruman, south-west Kenya. *Bull Entomol Res*. 91: 429-44. PubMed PMID: 11818038.

15.	Enyaru JC, Ouma JO, Malele II, Matovu E and Masiga DK, 2010. Landmarks in the evolution of technologies for identifying trypanosomes in tsetse flies. *Trends Parasitol*. 26: 388**–**394.

16.	Gikonyo NK, Hassanali A, Njagi PG, Saini RK, 2000. Behaviour of *Glossina morsitans morsitans* Westwood (Diptera: Glossinidae) on waterbuck *Kobus defassa* Ruppel and feeding membranes smeared with waterbuck sebum indicates the presence of allomones. *Acta Trop*. 77: 295-303. PubMed PMID: 11114392.

17.	Gikonyo NK, Hassanali A, Njagi PG, Gitu PM, Midiwo JO, 2002. Odor composition of preferred (buffalo and ox) and nonpreferred (waterbuck) hosts of some Savanna tsetse flies. *J Chem Ecol*. 28: 969-81. PubMed PMID: 12049234.

18.	Gikonyo NK, Hassanali A, Njagi PG, Saini RK, 2003. Responses of *Glossina morsitans morsitans* to blends of electroantennographically active compounds in the odors of its preferred (buffalo and ox) and nonpreferred (waterbuck) hosts. *J Chem Ecol*. 29: 2331-45. PubMed PMID: 14682515.

19.	Groenendijk, C.A.; Dekker, M.J.; Otieno, L.H.; Takken, W, 1993. A survey of the distribution of *Glossina pallidipes* Austen around the Lambwe Valley, South Nyanza District, Kenya. *Insect Sci Applic* 14: 225–228.

20.	Grootenhuis JG, Olubayo RO, 1993. Disease research in the wildlife-livestock interface in Kenya. *Vet Q*. 15: 55-9. PubMed PMID: 8372423.

21.	Hariyama T and Saini RK, 2001. Odour bait changes the attractiveness of colour for the tsetse fly. *Tropics* 10: 581-589.

22.	Hassanali A, McDowell PG, Owaga MA, Saini RK (1986) Identification of tsetse attractants from excretory products of a wild host animal, Syncerus caffer. *Insect Sci Applic* 7: 5-9.

23.	Imbuga, M.O.; Osir, E.O.; Labongo, V.L., 1992. Inhibitory effect of Trypanosoma brucei brucei on *Glossina morsitans* midgut trypsin in vitro. *Parasitol Res* 78: 273–276.

24.	Imbuga MO, Osir EO, Labongo VL, Darji N and Otieno LH, 1992. Studies on tsetse midgut factors that induce differentiation of blood-stream *Trypanosoma brucei brucei* in vitro. *Parasitol Res* 78: 10–15.

25.	Juri Ayub M, Smulski CR, Nyambega B, Bercovich N, Masiga D, Vazquez MP, Aguilar CF, Levin MJ, 2005. Protein-protein interaction map of the *Trypanosoma cruzi* ribosomal P protein complex. *Gene* 357: 129-36. PubMed PMID: 16120475.

26.	Jura WGZO, Otieno LH, 1994. Infectivity and virulence of Trypanosoma brucei metacyclics from Glossina morsitans morsitans salivary glands infected with tsetse DNA virus. *Insect Sci Applic* 15: 551–556.

27.	Kaaya GP, Ratcliffe A, Alemu P, 1986. Cellular and humoral defenses of Glossina Diptera: Glossinidae: reactions against bacteria, trypanosomes, and experimental implants. *J Med Entomol* 23: 30–43.

28.	Kaaya GP, Darji N, Otieno LH, 1987. Effects of bacteria, antibacterial compounds and trypanosomes on tsetse reproduction and longevity. *Insect Sci Applic* 8: 217–220.

29.	Kaaya GP and Darji N, 1988. The humoral defense system in tsetse: differences in response due to age, sex and antigen types. *Dev Comp Immunol*. 12: 255-68. PubMed PMID: 3384155.

30.	Kaddu JB and Mutinga J, 1988. Some concepts of the interaction of *Trypanosoma* (*Nannomonas*) *congolense* and *Glossina pallidipes*. *Ann Trop Med Parasitol.* 82: 229–234.

31.	Kaaya GP, 1989. A review of the progress made in recent years on research and understanding of immunity in insect vectors of human and animal diseases. *Insect Sci Applic* 10: 751–769.

32.	Kaaya GP and Munyinyi DM, 1995. Biocontrol potential of the entomogenous fungi *Beauveria bassiana* and *Metarhizium anisopliae* for tsetse flies (Glossina spp.) at developmental sites. *J Invertebr Pathol*. 66: 237-41. PubMed PMID: 8568279.

33.	Kaaya, GP, Mwangi EN, Ouna EA., 1996 Prospects for biological control of livestock ticks, Rhipicephalus appendiculatus and Amblyomma variegatum, using the entomogenous fungi Beauveria bassiana and Metarhizium anisopliae. *J Invertebr Pathol*. 67: 15-20. PMID: 8812559 [PubMed - indexed for MEDLINE]

34.	Kaaya, G.P. and Hassan, S. (2000) Entomogenous fungi as promising biopesticides for tick control. *Exp Appl Acarol*., 24: 913-926.

35.	Kitron, U., Otieno, L. H., Hungerford, L. L., Odulaja, A., Brigham, W. U. Okello, O. O., Joselyn, M., Mohamed-Ahmed M. M. and Cook, E. (2006). Spatial Analysis of the Distribution of Tsetse Flies in the Lambwe Valley, Kenya, Using Landsat TM Satellite Imagery and GIS. *Journal of Animal Ecology* 65: 371-380.

36.	Kokwaro, E.D., Otieno, L.H. , Chimtawi, M., 1991. Salivary glands of the tsetse *Glossina pallidipes* Austen infected with *Trypanosoma brucei* and virus particles: ultrastructural study. *Insect Sci Applic* 12: 661–669.

37.	Kongoro, J.A.; Osir, E.O.; Imbuga, M.O.; Oguge, N.O., 2002. Comparison of midgut trypsin / lectin activities and trypanosome infection rates in three Glossina species. *Insect Sci Applic* 22: 301.

38.	Leonard DE and Saini RK, 1994. Semiochemicals from anal exudate of larvae of tsetse flies *Glossina morsitans morsitans* Westwood and *Glossina morsitans centralis* Machado attract gravid females. *J. Chemical Ecol*. 19, 2039 – 2046.

39.	Mohamed-Ahmed MM and Odulaja A (1997). Diel activity patterns and host preferences of Glossina fuscipes fuscipes (Diptera:Glossinidae) along the shores of Lake Victoria, Kenya. *Bull Entomol Res* 87: 179-186. doi:10.1017/S0007485300027334.

40.	Manjiwa PAO and Otieno LH, 1990. Recombinant DNA probes reveal simultaneous infection of tsetse flies with different trypanosome species. *Mol Biochem Parasitol*, 40: 245–254.

41.	Malele II, Ouma JO, Enyaru JC, Matovu E, Alibu V, Auma JE, Onyoyo SG, Bateta R, Changasi RE, Mukiria PW, Ndung'u K, Gitonga PK, Mwaniki LM, Nyingilili HS, Lyaruu EA, Kapange LA, Kamau PK, Masiga DK. Comparative diagnostic and analytical performance of PCR and LAMP-based trypanosome detection methods estimated using pooled whole tsetse flies and midguts. *Vet Parasitol*. 2013 Jun 6. doi:pii: S0304-4017(13)00345-2. 10.1016/j.vetpar.2013.05.022. [Epub ahead of print] PubMed PMID: 23796572.

42.	Maniania NK, 1994 A laboratory technique for infecting adult tsetse with a fungal pathogen. *Insect Sci Applic* 5: 421–6.

43.	Maniania NK, 1998. A device for infecting adult tsetse flies, *Glossina* spp., with an entomopathogenic fungus in the field. *Biological Control* 11: 248-254.

44.	Maniania NK and Odulaja A, 1998. Effect of species, age, and sex of tsetse on response to infection by *Metarhizium anisopliae*. *Biocontrol*. 43: 311-323.

45.	Maniania NK and Nadel DJ, 1999. Tsetse’s lethal path. *Biocontrol News and Information.* 20, 7N-8N.

46.	Maniania NK, 2002. A low-cost contamination device for infecting tsetse flies, *Glossina* spp. with entomopathogenic fungus *Metarhizium anisopliae* in the field. *Biocontrol Science and Technology* 12, 59-66.

47.	Maniania NK, Okech MA, Adino JO, Opere JO, Ekesi S, 2004. Transfer of inoculum of *Metarhizium anisopliae* between adult *Glossina morsitans morsitans* and effects of fungal infection on blood feeding and mating behaviors. *J Pest Sci* 86:285-292.

48.	Maniania NK, Ekesi S, Odulaja A, Okech MA and Nadel DJ, 2006. Prospects of a fungus-contamination device for the control of tsetse fly *Glossina fuscipes fuscipes. Biocontrol Science and Technology* 16, 129-139.

49.	Maniania NK and Ekesi S, 2006. Tsetse’s lethal path (Part II). *Biocontrol News and Information.* 27, 17N-18N.

50.	Maniania, NK, Ekesi S, Odulaja A, Okech M A and Nadel DJ, 2006. Prospects of a fungus-contamination device for the control of tsetse fly *Glossina fuscipes fuscipes*. *Biocontrol Science and Technology*, *16*(2), 129-139.

51.	Maniania NK and Ekesi S, 2012. The use of entomopathogenic fungi in the control of tsetse flies. *J Invertebr Pathol*. 112 Suppl:S83-8. doi: 10.1016/j.jip.2012.07.019. Epub 2012 Jul 25. PMID: 22841947.

52.	Masiga DK, Ndung'u K, Tweedie A, Tait A, Turner CM, 2006. *Trypanosoma evansi*: genetic variability detected using amplified restriction fragment length polymorphism (AFLP) and random amplified polymorphic DNA (RAPD) analysis of Kenyan isolates. *Exp Parasitol*. 114: 147-53. PMID: 16624308.

53.	Masiga DK, Okech G, Irungu P, Ouma J, Wekesa S, Ouma B, Guya SO, Ndung'u JM, 2002. Growth and mortality in sheep and goats under high tsetse challenge in Kenya. *Trop Anim Health Prod*. 34: 489-501. PubMed PMID: 12537387.

54.	Mihok S, Olubayo RO, Darji N and Zweygarth E, 1993. The influence of host blood on infection rates in Glossina morsitans spp. infected with *Trypanosoma congolense*, *Trypanosoma brucei* and *Trypanosoma simiae*. *Parasitology* 107: 41–48.

55.	Mihok S, Munyoki EN, Masaninga F, Ndegwa PN, Olubayo, R.O., 1994. Isolation of Trypanosoma spp. from wild tsetse flies through procyclic expansion in Glossina morsitans centralis. *Acta Tropica*, 56, pp.p. 25–37.

56.	Mihok S, Maramba O, Munyoki E, Kagoiya J, 1995. Mechanical transmission of *Trypanosoma* spp. by African Stomoxyinae (Diptera: Muscidae). *Trop Med Parasitol*. 46:103-5. PubMed PMID: 8525279.

57.	Mihok S, 2002. The development of a multipurpose trap (the Nzi) for tsetse and other biting flies. *Bull Entomol Res* 92:385**–**403.

58.	Moloo SK, Kabata JM, Sabwa CL, 1994. A study on the maturation of procyclic Trypanosoma brucei brucei in Glossina morsitans centralis

59.	Muhigwa JB, Saini RK, Hassanali A, 1998. Effects of fly abundance on catch index of traps for *Glossina fuscipes fuscipes* (Diptera: Glossinidae). *J Med Entomol* 35: 148-52. PubMed PMID: 9538575.

60.	Mutero CM, Mutinga MJ, 1993. Defecation by Anopheles arabiensis mosquitoes of host blood infected with live *Trypanosoma congolense*. *Trop Med Parasitol*. 44: 23-6. PubMed PMID: 8516628.

61.	Muturi CN, Ouma JO, Malele II, Ngure RM, Rutto JJ, et al. (2011) Tracking the Feeding Patterns of Tsetse Flies (Glossina Genus) by Analysis of Bloodmeals Using Mitochondrial Cytochromes Genes. *PLoS ONE* 6(2): e17284. doi:10.1371/journal.pone.0017284

62.	Ng'ayo MO, Njiru ZK, Kenya EU, Muluvi GM, Osir EO, Masiga DK, 2005. Detection of trypanosomes in small ruminants and pigs in western Kenya: important reservoirs in the epidemiology of sleeping sickness? *Kinetoplastid Biol Dis*. 14;4:5. PubMed PMID: 16018802; PubMed Central PMCID: PMC1200563.

63.	Nguu EK, Osir EO, Imbuga MO, Olembo NK, 1996. The effect of host blood in the in vitro transformation of bloodstream trypanosomes by tsetse midgut homogenates. *Med Vet Entomol* 10: 317–322.

64.	Njagu Z, Mihok S, Kokwaro E, Verloo D, 1999. Isolation of *Trypanosoma brucei* from the monitor lizard (*Varanus niloticus*) in an endemic focus of Rhodesian sleeping sickness in Kenya. *Acta Trop*. 72: 137-48. PubMed PMID: 10206114.

65.	Njiru ZK, Constantine CC, Masiga DK, Reid SA, Thompson RC, Gibson WC, 2006. Characterization of *Trypanosoma evansi* type B. *Infect Genet Evol.* 6: 292-300. PubMed PMID: 16157514.

66.	Nyeko JH, Golder TK, Otieno LH, 1988. Selection for drug resistance in *Trypanosoma congolense* during cyclic transmissions through *Glossina morsitans morsitans* and drug treated rabbits. *Acta Trop*. 45: 21-6. PubMed PMID: 2896442.

67.	Nyeko JHP, Golder TK, Otieno LH, Ssenyonga GSZ, 1989. *Trypanosoma congolense*: Drug resistance during cyclical transmissions in tsetse flies and syringe passages in mice. *Exp Parasitol* 69: 357–362.

68.	Nyindo M, Chimtawi M and Owor J, 1980. Fine sructure of metacyclic forms of Trypanosoma brucei grown in continuous culture. *Am J Trop Med Hyg* 29: 774–778.

69.	Nyindo M and Rurangirwa R, 1981. *Trypanosoma brucei*: continous cultivation of antigenically stable parasites at 290C and induction of antigenic variants at 370C. *Insect Sci Applic* 2: 263–266.

70.	Nyindo, M, Essuman S, Chesang R, Muteria P, 1996. Immunity induced by vaccination with *Rhipicephalus appendiculatus* salivary gland antigens does not augment protective immunity acquired naturally by exposing rabbits to adult ticks. *J Med Entomol*. 33: 559-65. PMID: 8699449

71.	Odulaja, A. and Mohamed-Ahmed MM, 1997. Estimation of the efficiency of the biconical trap for *Glossina fuscipes fuscipes* along the lake Victoria shore, Kenya. *Entomologia Experimentalis et Applicata*, 82: 19–24. doi: 10.1046/j.1570-7458.1997.00109.x

72.	Odulaja A and Abu-Zinid IM, 1997. The relative efficiencies of Latin square and randomized complete block designs for insect trapping experiments: an investigation using field data on tsetse flies. *Ecological Entomology* 22: 184-188.

73.	Odulaja A and Chuka Madubunyi L, 1997. A sampling bias model for odour-baited traps in relation to tsetse hunger cycle and population suppression. *Ecological Modelling*, *104*(2-3), 165-173

74.	Odulaja, A., Mihok, S., & Abu-Zinid, I. M. (1998). The magnitude of site and time interaction effect in tsetse fly (Diptera: Glossinidae) trap catches. *Bull Entomol Res* 88: 59-64.

75.	Odulaja A, Baumgärtner J, Mihok S, Abu-Zinid IM, 2001. Spatial and temporal distribution of tsetse fly trap catches at Nguruman, southwest Kenya. *Bull Entomol Res*. 91: 213-20. PubMed PMID: 11415476.

76.	Ohaga SO, Kokwaro ED, Ndiege IO, Hassanali A, Saini RK, 2007. Livestock farmers' perception and epidemiology of bovine trypanosomosis in Kwale District, Kenya. *Prev Vet Med*. 80: 24-33. PubMed PMID: 17324481.

77.	Omolo EO, James MD, Osir EO, 1997. Thomson JA. Cloning and expression of a *Bacillus thuringiensis* (L1-2) gene encoding a crystal protein active against *Glossina morsitans morsitans* and *Chilo partellus*. *Curr Microbiol*. 34: 118-21. PubMed PMID: 9003589.

78.	Omolo MO, Hassanali A, Mpiana S, Esterhuizen J, Lindh J, Lehane MJ, Solano P, Rayaisse JB, Vale GA, Torr SJ, Tirados I, 2009. Prospects for developing odour baits to control *Glossina fuscipes* spp., the major vector of human African trypanosomiasis. *PLoS Negl Trop Dis*. 3(5):e435. doi: 10.1371/journal.pntd.0000435.

79.	Osir EO, Abubakar L and Imbuga MO, 1995. Purification and characterization of a midgut lectin-trypsin complex from the tsetse fly *Glossina longipennis*. *Parasitol Res* 81, 276–281.

80.	Otieno LH and Darji N, 1987. The investigation of *Trypanosoma brucei* isolates obtained from *Glossina pallidipes* in South Nyanza, Kenya. *J Trop Med Hyg*. 90: 259-63. PubMed PMID: 3669129.

81.	Otieno LH, 1978. The presence in salivary secretions of *Glossina morsitans morsitans* of stages of *Trypanosoma* (*Trypanozoon*) *brucei* other than occurring in the salivary glands. *Transactions of the Roy Soc Trop Med Hyg* 72: 622–626.

82.	Otieno LH, Darji N, Onyango P, 1976. Development of *Trypanosoma* (*Trypanozoon*) *brucei* in Glossina morsitans inoculated into the tsetse haemocoel. *Acta Tropica Separatum*, 33: 144–150.

83.	Otieno LH, Darji N and Onyango P, 1990. Electrophoretic analysis of Trypanosoma brucei sub-group stocks from cattle, tsetse and patients from Lambwe Valley, Western Kenya. *Insect Sci Applic* 11: 281–287.

84.	Otieno LH and Darji PG, 1977. An assessment of trypanosome infection of wild *Glossina pallidipes* Austen using fly dissection salivation and mouse inoculation methods. In *In: Proceedings 15th ISCTRC Conference*. Gambia: ISCTRC, pp. 273 – 278.

85.	Otieno LH, Darji PG, Olobo J and Onyango P, 1980. Efficiency of wild *Glossina pallidipes* Austen as vectors of pathogenic African trypanosomes. *In: Isotope and Radiation Research on Animal Diseases and their Vectors*, p.In: Isotope and Radiation Research on Animal Diseases and their Vectors.

86.	Otieno LH, Darji PG, Onyango P and Mpanga E, 1983. Some observations on factors associated with the development of *Trypanosoma brucei brucei* infections in *Glossina morsitans morsitans*. *Acta Tropica*, 40: 113–120.

87.	Otieno LH and Darji PG, 1985. Characterization of potentially man-infective *Trypanosoma brucei* from an endemic area of sleeping sickness in Kenya. *Trop Med Parasitol* 36: 123–126.

88.	Otieno LH, 1979. The abundance of pathogenic African trypanosomes in the salivary secretions of wild *Glossina pallidipes*. *Annals of Tropical Medicine and Parasitology*, 73, p.P. 583–588.

89.	Owaga MLA, 1981. Ecological studies and laboratory rearing of the tsetse species *Glossina longipennis* Corti in Kenya. *Insect Sci Applic* 2: 97–200.

90.	Owaga MLA, 1984. Preliminary observations on the efficacy of olfactory attractants derived from wild hosts of tsetse. *Insect Sci Applic* 5: 87-90.

91.	Owaga MLA, 1985. Observations on the efficacy of buffalo urine as a potent olfactory attractant for *Glossina pallidipes* Austen. *International Journal of Tropical Insect Science* 6.05 (1985): 561-566.

92.	Owaga MLA and Challier A, 1985. Catch composition of the tsetse Glossina pallidipes Austen in revolving and stationary traps with respect to age, sex ratio and hunger stage. *International Journal of Tropical Insect Science* 6: 711-718.

93.	Owaga MLA, Hassanali A and McDowell PG, 1988. The role of 4-cresol and 3-n-propylphenol in the attraction of tsetse flies to buffalo urine. *Insect Science and its Application* 9: 95-100.

94.	Patel NY, Youdeowei and Odhiambo TR, 1981. The composition of the salivary gland secretion of the tsetse, *Glossina morsitans morsitans* Westwood 1850 (Diptera: Glossinidae). *Insect Sci Applic* 1: 383–387.

95.	Pickett JA, Birkett MA, Dewhirst SY, Logan JG, Omolo MO, Torto B and Leal WS, 2010. Chemical ecology of animal and human pathogen vectors in a changing global climate. *J Chem Ecol* 36: 113-121.

96.	Randolph SE, Dransfield RD, Rogers DJ, 1989. Effect of host odours on trap catch composition of *Glossina pallidipes* in Kenya. *Med Vet Entomol*. 3: 297-306. PubMed PMID: 2519676.

97.	Saini RK, 1981. The pattern of sound production by the tsetse fly : *Glossina morsitans* m*orsitans* West, 1850 (Diptera, Glossinidae). *Insect Sci. Applic*. 1: 167-169.

98.	Saini RK, 1981. Effects of age and hunger on the pattern of sound production in the tsetse *Glossina morsitans morsitans* West. 1850 (Diptera, Glossinidae). *Insect Sci. Applic*. 1: 393-397.

99.	Saini RK, 1983. Sound production associated with feeding behaviour of the tsetse, *Glossina morsitans morsitans*. Ent Exp and Appl 34, 1 - 8.

100.	Saini RK, 1984. Acoustic emissions of tsetse. *Insect Sci. Applic*. 5, 341-344.

101.	Saini RK, 1985. Sound production associated with sexual behaviour of the tsetse, *Glossina morsitans morsitans. Insect Sci. Applic*. 6: 637-644.

102.	Saini RK, 1986. Antennal responses of *Glossina morsitans morsitans* to buffalo urine - a potent olfactory attractant of tsetse flies. *Insect Sci. Applic*. 7: 771-775.

103.	Saini RK and Dransfield RD, 1987. A behavioural bioassay to identify attractive odours for Glossinidae. *Med Vet Entomol*. 1: 313-8.

104.	Saini RK, Hassanali A and Dransfield RD,1989. Antennal responses of tsetse to analogues of the attractant 1-octen-3-o1. *Physiol. Ent*. 14: 85 - 90.

105	Saini RK, 1990. Responses of tsetse flies *Glossina* spp. (Diptera: Glossinidae) to phenolic kairomones in a wind tunnel. *Insect Sci. Applic*. 11: 369 - 375.

106.	Saini RK, 1992. Olfactory sensitivity of tsetse flies to phenolic kairomones. *Insect Sci. Applic*. 13: 95 – 104.

107.	Saini RK, Hassanali A, Andoke J., Ahuya P. and Ouma WP, 1996. Identification of the major components of the larviposition pheromone from the larvae of tsetse flies *Glossina morsitans morsitans* Westwood and *Glossina morsitans centralis* Machado J*. Chemical Ecol*. 22, 1211 – 1220.

108.	Saini RK, Hassanali A, Ahuya P, Andoke J and Nyandat E (1993). Close range responses of tsetse flies *Glossina morsitans morsitans* Westwood (Diptera: Glossinidae) to host body kairomones. *Discovery & Innovation* 5, 149 - 153

109.	Saini RK, Hassanali A, 2007. A 4-alkyl-substituted analogue of guaiacol shows greater repellency to savannah tsetse (Glossina spp.). *J Chem Ecol*. 33: 985-95. PubMed PMID: 17404820.

110.	Snow WF, Tarimo A, 1985. Observations on age-grouping the tsetse fly, Glossina pallidipes, by wing-fray and ovarian dissection, on the south Kenya Coast. *Ann Trop Med Parasitol* 79: 457–461.

111.	Tarimo N, Njau BC, Otieno LH, 1991. Epizootiology of trypanosomiasis in Lambwe Valley, Kenya, East Africa. *Insect Sci Applic* 12: 379–384.

112.	Tait A, Macleod A, Tweedie A, Masiga D, Turner CM, 2006. Genetic exchange in *Trypanosoma brucei*: evidence for mating prior to metacyclic stage development. *Mol Biochem Parasitol* 151: 133-6. PMID: 17134768.

113.	Tarimo SA, Snow FW, Butler L, Dransfield R, 1985. The probability of tsetse acquiring trypanosome infection from single blood meal in different localities in Kenya. *Acta Trop*. 42: 199-207. PMID: 2865875.

114.	Tikubet G, Dransfield RD, Otieno RD, Morgan H, 1991. Direct estimation of tsetse “challenge” and trypanosomiasis “risk.” In ISTRC. ISTRC, pp. 168–173.

115.	Thuita JK, Kagira JM, Mwangangi D, Matovu E, Turner CM, Masiga D, 2008. *Trypanosoma brucei rhodesiense* transmitted by a single tsetse fly bite in vervet monkeys as a model of human African trypanosomiasis. *PLoS Negl Trop Dis*. 2(5):e238. doi: 10.1371/journal.pntd.0000238. PubMed PMID: 18846231

116.	Williams B, Dransfield R, Brightwell R, 1990. Monitoring tsetse fly populations. I. The intrinsic variability of trap catches of *Glossina pallidipes* at Nguruman, Kenya. *Med Vet Entomol*. 4: 167-79. PubMed PMID: 2132981.

117.	Williams B, Brightwell R, Dransfield R, 1990. Monitoring tsetse fly populations. II. The effect of climate on trap catches of Glossina pallidipes. *Med Vet Entomol*. 4: 181-93. PubMed PMID: 2132982.

118.	Youdeowei A, 1976. Salivary secretion in wild Glossina pallidipes Austen. (Diptera, Glossinidae). *Acta Tropica Separatum*, 33: 369–375.

##  Leishmaniasis

119.	Basimike M, Mutinga MJ, Kumar R, 1991. Distribution of sandflies (Diptera: Psychodidae) in three vegetation habitats in the Marigat area, Baringo District, Kenya. *J Med Entomol*. 28: 330-3. PubMed PMID: 1875361.

120.	Basimike M and Mutinga MJ, 1995. Effects of permethrin-treated screens on phlebotomine sand flies, with reference to Phlebotomus martini (Diptera: Psychodidae). *J Med Entomol*. 32: 428-32. PubMed PMID: 7650702.

121.	Basimike M and Mutinga MJ, 1997. Studies on the vectors of leishmaniasis in Kenya: phlebotomine sandflies of Sandai location, Baringo district. *East Afr Med J*. 74: 582-5. PubMed PMID: 9487436.

122.	Basimike M, Mutinga MJ, Mutero CM, 1989. Vertical Distribution of Phlebotomine Sandflies in Two Habitats in Marigat Leishmaniasis Endemic Focus, Baringo District, Kenya. Insect Sci Applic 10(5).

123.	Beach R, Young DG, Mutinga MJ, 1983. New phlebotomine sand fly colonies: rearing Phlebotomus martini, *Sergentomyia schwetzi*, and *Sergentomyia africana* (Diptera: Psychodidae). *J Med Entomol*. 20: 579-84. PubMed PMID: 6644755.

124.	Beach R, Young DG, Mutinga MJ, 1982. *Phlebotomus* (Phlebotomus) *duboscqi* from Kenya: a new record. *Trans R Soc Trop Med Hyg*. 76: 707-8. PubMed PMID: 7179431.

125.	Gebre-Michael T, Lane RP, 1996. A new sandfly species, *Phlebotomus* (*Larroussius*) *ashfordi* (Diptera, Psychodidae) from Ethiopia, previously confused with *P.* (*L.*). *aculeat*us. *Ann Trop Med Parasitol*. 90: 523-31. PubMed PMID: 8915129.

126.	Irungu LW, Mutinga MJ and Kokwaro ED, 1986. Chorionic sculpturing of eggs of some Kenyan phlebotomine sandflies. *International Journal of Tropical Insect Science*, 7: 45-48.

127.	Killick-Kendrick R, Killick-Kendrick M, Tang Y, Sang DK, Johnson RN, Ngumbi PM, 1993. Phlebotomine sandflies of Kenya (Diptera: Psychodidae). I. The validity of *Phlebotomus* (*Larroussius*) *elgonensis* Ngoka, Madel and Mutinga. *Ann Trop Med Parasitol*. 1993 87: 207-15. PubMed PMID: 8561529.

128.	Mahamat H, Hassanali A, 1998. Cuticular hydrocarbon composition analysis for taxonomic differentiation of phlebotomine sandfly species (Diptera: Psychodidae) in Kenya. *J Med Entomol*. 35: 778-81. PubMed PMID: 9775608

129.	Massamba NN, Mutinga MJ, Kamau CC, 1998. Characterisation of Leishmania isolates from Laikipia District, Kenya. *Acta Trop*. 713):293-303. PubMed PMID:

130.	Massamba NN and Mutinga MJ, 1992. Recombinant kinetoplast DNA (kDNA) probe for identifying *Leishmania tropica*. *Acta tropica*, 52: 1-15.

131.	Mutero CM, Mutinga MJ, Amimo FA, 1993. Gonotrophic status of female phlebotomine sandflies collected by three different methods in Kenya. *Med Vet Entomol*. 7: 291-3. PubMed PMID: 8369566.

132.	Mutero CM, Mutinga MJ, Ngindu AM, Kenya PR, Amimo FA, 1992. Visceral leishmaniasis and malaria prevalence in West Pokot District, Kenya. *East Afr Med J*. 69: 3-8. PMID: 1628545

133.	Mutero MK, Kamau CC, Basimike M, Mutero CM, Kyai FM, 1992. Visceral leishmaniasis and malaria prevalence in West Pokot District, Kenya. *East Afr Med J*. 69: 9-13. PMID: 1628553.

134.	Mutero CM, Mutinga MJ, Birley MH, Amimo FA and Munyinyi DM, 1991. Description and Performance of an Updraft Trap for Sandflies. Trop Med Parasitol 42: 407.

135.	Mutinga MJ, Basimike M, Kamau CC, Mutero CM, 1990. Epidemiology of leishmaniases in Kenya. Natural host preference of wild caught phlebotomine sandflies in Baringo District, Kenya. *East Afr Med J*. 67: 319-27. PubMed PMID: 2390954.

136.	Mutinga MJ, Kamau CC, Basimike M, Mutero CM, Kyai FM, 1992. Studies on the epidemiology of leishmaniasis in Kenya: flight range of phlebotomine sandflies in Marigat, Baringo District. *East Afr Med J*. 69: 9-13. PubMed PMID: 1628553.

137.	Mutinga MJ, Kamau CC, Mwandandu J, 1987. Laboratory investigations on the survival and fecundity of *Phlebotomus duboscqi* (Diptera, Psychodidae), a vector of Leishmania major in Kenya. *Trop Med Parasitol*. 38: 86-8. PubMed PMID: 3629141.

138.	Mutinga MJ, Kyai FM, 1985. A new record of Sergentomyia garnhami (Heisch, Guggisberg and Teesdale, 1956) in Kenya. *Trans R Soc Trop Med Hyg*. 79: 137-8. PubMed PMID: 3992637.

139.	Mutinga MJ, Massamba NN, Basimike M, Kamau CC, Amimo FA, Onyido AE, Omogo DM, Kyai FM, Wachira DW, 1994. Cutaneous leishmaniasis in Kenya: *Sergentomyia garnhami* (Diptera Psychodidae), a possible vector of *Leishmania major* in Kitui District: a new focus of the disease. *East Afr Med J*. 71: 424-8. PubMed PMID: 7828493.

140.	Mutinga MJ, Ngoka JM, 1978. Incrimination of the vector of visceral leishmaniasis in Kenya. *East Afr Med J*. 55: 337-40. PubMed PMID: 699848.

141.	Mutinga MJ, Renapurkar DM, Wachira DW, Basimike M, Mutero CM, 1993. A bioassay to evaluate the efficacy of permethrin-impregnated screens used against phlebotomine sandflies (Diptera: Psychodidae) in Baringo District of Kenya. *East Afr Med J*. 70: 168-70. PubMed PMID: 8261945.

142.	Mutinga MJ, 1991. A review of investigations on leishmaniasis vectors in Kenya. *Parassitologia*. 33 Suppl:453-61. Review. PubMed PMID: 1841244.

143.	Mutinga MJ, 1971. *Phlebotomus longipes*, as a vector of cutaneous leishmaniasis in Kenya. *Trans R Soc Trop Med Hyg*. 65: 106. PubMed PMID: 5092405.

144.	Mutinga MJ, 1975. Phlebotomus fauna in the cutaneous leishmaniasis focus of Mt. Elgon, Kenya. *East Afr Med J*. 52: 340-7. PubMed PMID: 1192986.

145.	Mutinga MJ, 1975. The animal reservoir of cutaneous leishmaniasis on Mount Elgon, Kenya. *East Afr Med J*. 52: 142-51. PubMed PMID: 1140141.

146.	Mutinga MJ, 1981. An Efficient Trap for Sandflies (Diptera, Phlebotomidae). *Insect Sci Applic* 1: 203-206.

147.	Mutinga, Kaddu, Olobo, Kyai, Omogo PG Medical Vectors, and ICIPE. Studies on Vectors of Kala-azar in Kenya: *Phlebotomus Heischi* (Diptera, Psychodidae) Kirk and Lewis, a New Male Record in Kenya. *Insect Sci Applic* 4: 253-254, 1983.

148.	Mutinga, Kamau PG Medical Vectors, and ICIPE. Investigations of the Epidemiology of Leishmaniases in Kenya II. The Breeding Sites of Phlebotomine Sandflies in Marigat, Baringo District. *Insect Sci Applic* 7(1); 37-44, 1986.

149.	Mutinga, Kyai, Kamau, Omogo PG Medical Vectors, and ICIPE. Epidemiology of Leishmaniasis in Kenya - III. Host Preference Studies Using Various Types of Animal Baits at Animal Burrows in Marigat, Baringo District. *Insect Sci Applic* 7: 191-197, 1986.

150.	Mutinga MJ, 1996. Phlebotomine sandflies (Diptera:, Psychodidae): their importance and some aspects of control. *East Afr Med J*. 73: 48-9. Review. PubMed PMID: 8625863.

151.	Mutinga, Kyai, and Omogo PG, 1986. Medical Vectors. Investigations on the Epidemiology of Leishmaniases in Kenya - I. Studies on Vectors of *Leishmania* Major in Marigat, Baringo District, Kenya. *Insect Sci Applic* 7(2); 181-189.

152.	Mutinga MJ, Basimike M, Kamau CC, Mutero CM, 1990. Epidemiology of Leishmaniases in Kenya. Natural Host Preference of Wild Caught Phlebotomine Sandflies in Baringo District, Kenya. *East Afr Med J* 67(5), 1990.

153.	Mutinga MJ, Basimike M, Mutero CM and Ngindu AM, 1992. The Use of Permethrin-impregnated Wall Cloth (Mbu Cloth)for the Control of Vectors of Malaria and Leishmaniases in Kenya-11.Effect on Phlebotomine Sandfly Populations. Insect Sci Applic 13(2).

154.	Mutinga MJ and Ngoka PG, 1981. Medical Vectors. Suspected Vectors of Lizard Leishmaniasis in Kenya and Their Possible Role in Partial Immunization of the Human Population Against *Leishmania Donovani* in Kala-azar Endemic Areas. Insect Sci Applic 1(2): 207-210.

155.	Mutinga MJ and Odhiambo PG, 1982. Medical Vectors, and ICIPE. Studies on Infection Rates of Human-baited Anthropophilic Sandflies in Machakos District, Kenya. Insect Sci Applic 3: 211-214,.

156.	Mutinga MJ, Kaddu, Olobo, Kyai, Omogo PG, 1983. Medical Vectors, and ICIPE. Studies on Vectors of Kala-azar in Kenya: *Phlebotomus* Heischi (Diptera, Psychodidae) Kirk and Lewis, a New Male Record in Kenya. *Insect Sci Applic* 4: 253-254.

157.	Ngoka JM, Madel G, Mutinga MJ, 1975. *Phlebotomus* (*Larroussius*) *elgonensis* sp. nov. (Diptera, Phlebotomidae), a new sandfly from Kenya. *East Afr Med J*. 52: 132-41. PubMed PMID: 1140140.

158.	Ngumbi PM, Irungu LW, Ndegwa PN and Maniania NK, 2011. Pathogenicity of *Metarhizium anisopliae* (Metch) Sorok and *Beauveria bassiana* (Bals) Vuill to adult *Phlebotomus duboscqi* (Neveu-Lemaire) in the laboratory. *J Vector Borne Dis* 48: 37–40.

159.	Ngumbi PM, Irungu LW, Robert LL, Gordon DM, Githure JI, 1998. Abundance and nocturnal activities of phlebotomine sandflies (Diptera: Psychodidae) in termite hills and animal burrows in Baringo district, Kenya. *African Health Sci* 5: 28–34.

160.	Ngumbi PM, Kaburi JC, Anjili CO and Haas F, 2010. *Phlebotomus* (*Larroussius*) *orientalis* (Diptera: Psychodidae) as a probable secondary vector of visceral leishmaniasis in Kenya. *J Vector Borne Dis* 47: 58-60.

161.	Onyido AE and Mutinga MJ, 1995. Ecology of *Sergentomyia garnhami*, (Diptera:Psychodidae) in a kala-azar endemic area of Tseikuru, Kitui District, Kenya. I: The breeding sites. *East Afr Med J*. 72: 83-8. PubMed PMID: 7796760.

162.	Onyido AE and Mutinga MJ, 1995. Ecology of *Sergentomyia garnhami*, (Diptera:Psychodidae) in a kala-azar endemic area of Tseikuru, Kitui District, Kenya. II: Day resting sites. *East Afr Med J*. 2: 89-93. PubMed PMID: 7796761.

163.	Rogo LM, Kokwaro ED, Mutinga MJ, Khamala CP, 1992. Differentiation of vector species of phlebotominae (Diptera: Psychodidae) in Kenya by chorionic culpturing of their eggs. *J Med Entomol*. 29: 1042-4. PubMed PMID: 1460620.

164.	Schlein Y and Jacobson RL, 1996. *Why Is Man Unsuitable Reservoir for the Transmission of Leishmania Major?* *Experimental Parasitol* 28, 1996.

## Arboviruses

165.	Beaty BJ, 2005. *Control of Arbovirus Diseases: Is the Vector the Weak Link?* Archives of Virology 19.

166.	Lwande, OW, Irura Z, Tigoi C, Chepkorir E, Orindi B, Musila L, Venter M, Fischer A and Sang R, 2012. Seroprevalence of Crimean Congo Hemorrhagic Fever Virus in Ijara District, Kenya. *Vector Borne Zoonotic Dis* 12: 727-32. DOI: 10.1089/vbz.2011.0914

167.	Masembe C, Michuki G, Onyango M, Rumberia C, Bishop RP, Djikeng A, Kemp SJ, Orth A, Skilton R, Stahl K and Fischer A, 2012. Viral metagenomics demonstrates that domestic pigs are a potential reservoir for Ndumu virus. *J. Virol*. 9, 218.

168.	Muriu, S.M., E. J. Muturi, J. I. Shililu, C. M. Mbogo, J. M. Mwangangi, B. G. Jacob, L. W. Irungu, et al. *Host Choice and Multiple Blood Feeding Behaviour of Malaria Vectors and Other Anophelines in Mwea Rice Scheme, Kenya*. Malaria Journal 7:43, 2008.

169.	Mutinga MJ, Renapurkar DM, Wachira DW, Mutero CM, Basimike M, 1992. Evaluation of the residual efficacy of permethrin-impregnated screens used against mosquitoes in Marigat, Baringo district, Kenya. *Trop Med Parasitol*. 43: 277-81. PMID: 1363445

170.	Mwangangi JM, Midega J, Kahindi S, Njoroge J, Nzovu J. Githure J, Mbogo CM, Beier J, 2012. Mosquito Species Abundance and Diversity in Malindi, Kenya and Their Potential Implication in Pathogen Transmission. Parasitology Res 110.

171.	Tchouassi et al.: Trapping of Rift Valley Fever (RVF) vectors using Light Emitting Diode (LED) CDC traps in two arboviral disease hot spots in Kenya. *Parasites & Vectors* 2012 5:94.

172.	Tchouassi DP, Sang R, Sole CL, Bastos ADS, Mithoefer K, et al. (2012) Sheep Skin Odor Improves Trap Captures of Mosquito Vectors of Rift Valley Fever. *PLoS Negl Trop Dis* 6(11): e1879. doi:10.1371/journal.pntd.0001879

173.	[Tchouassi DP](http://www.ncbi.nlm.nih.gov/pubmed?term=Tchouassi DP%5BAuthor%5D&cauthor=true&cauthor_uid=23326620), [Sang R](http://www.ncbi.nlm.nih.gov/pubmed?term=Sang R%5BAuthor%5D&cauthor=true&cauthor_uid=23326620), [Sole CL](http://www.ncbi.nlm.nih.gov/pubmed?term=Sole CL%5BAuthor%5D&cauthor=true&cauthor_uid=23326620), [Bastos AD](http://www.ncbi.nlm.nih.gov/pubmed?term=Bastos AD%5BAuthor%5D&cauthor=true&cauthor_uid=23326620), [Teal PE](http://www.ncbi.nlm.nih.gov/pubmed?term=Teal PE%5BAuthor%5D&cauthor=true&cauthor_uid=23326620), [Borgemeister C](http://www.ncbi.nlm.nih.gov/pubmed?term=Borgemeister C%5BAuthor%5D&cauthor=true&cauthor_uid=23326620), [Torto B](http://www.ncbi.nlm.nih.gov/pubmed?term=Torto B%5BAuthor%5D&cauthor=true&cauthor_uid=23326620). (2013). Common host-derived chemicals increase catches of disease-transmitting mosquitoes and can improve early warning systems for Rift Valley fever virus. *PLoS Negl Trop Dis*. 2013;7(1):e2007. doi: 0.1371/journal.pntd.0002007. Epub 2013 Jan 10.

## Ticks

## 174. Dossa SC, Kaaya GP, Essuman S, Odulaja A and Assoku RGK, 1996. Acquisition of resistance to the tick in Boran cattle, *Bos indicus* and the effects of *Trypanosoma congolense* and *Babesia bigemina* on host resistance. *Vet Parasitol* 62: 317-330.

## 175. Hassan SM, Dipeolu OO, Malonza MM, 1994. Natural attraction of livestock ticks by the leaves of a shrub. *Trop Anim Health Prod*. 26: 87-91. PMID: 7941035

176.	Kaaya GP and Hassan S, 2000. Entomogenous fungi as promising biopesticides for tick control. *Experimental and Applied Acarology*, 24, 913-926.

177.	Kaaya GP, Mwangi EN, Ouna EA, 1996. Prospects for biological control of livestock ticks, *Rhipicephalus appendiculatus* and *Amblyomma variegatum*, using the entomogenous fungi Beauveria bassiana and Metarhizium anisopliae. *J Invertebr Pathol*. 67: 15-20. PMID: 8812559

178.	Nana P, Maniania NK, Maranga RO, Kutima HL, Boga HI, Nchu F, Eloff JN, 2010. Attraction response of adult *Rhipicephalus appendiculatus* and *Rhipicephalus pulchellus* (Acari: Ixodidae) ticks to extracts from Calpurnia aurea (Fabaceae). *Vet Parasitol*. 174: 124-30. doi: 10.1016/j.vetpar.2010.08.016.

179.	Nana P., Maniania N.K., Maranga R.O., Boga H.I., Kutima H.L. and Eloff J.N., 2011. [Compatibility between *Calpurnia aurea* leaf extract, attraction aggregation, and attachment pheromone and entomopathogenic fungus *Metarhizium anisopliae* on viability, growth, and virulence of the pathogen](http://www.springerlink.com/content/4w6v502kh576372l/). *Journal of Pest Science.* DOI 10.1007/s10340-011-0399-5.

180.	Nchu F, Maniania NK, Hassanali A, Eloff JN, 2010. Performance of a *Metarhizium anisopliae*-treated semiochemical-baited trap in reducing *Amblyomma variegatum* populations in the field. *Vet Parasitol*. 169: 367-72. doi: 10.1016/j.vetpar.2010.01.020. PMID: 20153931

181.	Nchu F, Maniania NK, Touré A, Hassanali A and Eloff JN, 2009. The use of a semiochemical bait to enhance exposure of *Amblyomma variegatum* (Acari: Ixodidae) to *Metarhizium anisopliae* (Ascomycota: Hypocreales). *Vet. Parasitol*. 160, 279-284.

182.	Nchu F, Maniania NK, Hassanali A, Eloff KN, 2010. Optimizing modes of inoculation of *Rhipicephalus* ticks (Acari: Ixodidae) with a mitosporic entomopathogenic fungus in the laboratory. *Exp Appl Acarol*. 51: 373-82. doi: 10.1007/s10493-009-9330-7. PMID: 20082120

184.	Nyindo M, Essuman S, Chesang R, Muteria P, 1996. Immunity induced by vaccination with Rhipicephalus appendiculatus salivary gland antigens does not augment protective immunity acquired naturally by exposing rabbits to adult ticks. *J Med Entomol*. 33: 559-65. PMID: 8699449
